# Atypical Presentation of Pheochromocytoma: A Case Report of Ventricular Tachycardia in a Young Woman

**DOI:** 10.7759/cureus.70405

**Published:** 2024-09-28

**Authors:** Gurjot Singh, Kanishka Goswami, Kanwarpreet S Sandhu, Anupam Singh, Shubam Trehan, Didar Singh

**Affiliations:** 1 Internal Medicine, Springfield Memorial Hospital, Springfield, USA; 2 Hospital Medicine, Springfield Clinic, Springfield, USA; 3 Internal Medicine, Springfield Memorial Hospital, Southern Illinois University, Springfield, USA; 4 Internal Medicine, Saint John's Hospital, Southern Illinois University, Springfield, USA

**Keywords:** adrenal mass, catecholamine hypersecretion, neuroendocrine tumor, palpitations, pheochromocytoma, ventricular tachycardia, ventricular tachycardia in young female

## Abstract

Venturing beyond typical arrhythmias, a case of ventricular tachycardia (VT) in a 28-year-old woman who was initially presenting as benign tachycardia took an unexpected turn as her palpitations evolved to include severe sweating and significant weight loss. Initially, her symptoms like palpitations were treated with metoprolol, and given her age and lack of significant risk factors, the tachycardia was considered benign. However, as time passed, she developed severe sweating and noticeable weight loss, prompting a deeper investigation. This thorough exploration uncovered a 4 cm adrenal mass, which turned out to be pheochromocytoma, a rare tumor that can present with atypical symptoms. Initial diagnoses considered a range of conditions, from idiopathic VT and structural heart disease to electrolyte imbalances and thyroid disorders. Each possibility was carefully evaluated and ruled out, leading to the discovery of elevated plasma free metanephrines and imaging findings that confirmed pheochromocytoma. The successful diagnosis of this case demonstrates the importance of keeping rare conditions like pheochromocytoma in mind, even when initial symptoms suggest a different diagnosis. It serves as a reminder of the value of a comprehensive diagnostic approach and the need to stay vigilant for less common causes behind seemingly straightforward symptoms.

## Introduction

Pheochromocytoma is a rare, life-threatening tumor that originates from the adrenal medulla and secretes excess catecholamines, leading to a variety of symptoms that can resemble more common conditions [1-2]. The diagnosis of this tumor can be particularly challenging due to its nonspecific and variable presentation, especially in young patients where benign arrhythmias are typically suspected [3-4]. Differential diagnoses for palpitations in this population typically include idiopathic ventricular tachycardia (VT), anxiety or panic disorders, electrolyte imbalances, hyperthyroidism, and structural heart disease [5-6]. This case report discusses the difficulties in diagnosing pheochromocytoma in a 28-year-old woman who presented with intermittent palpitations, a symptom often linked to benign causes in healthy young individuals.

Despite initial treatment for what was thought to be benign arrhythmias, the patient’s condition worsened, presenting with severe sweating and substantial weight loss. A comprehensive diagnostic evaluation identified elevated levels of plasma free metanephrines and an adrenal mass, leading to the diagnosis of pheochromocytoma. This case highlights the necessity of maintaining a broad differential diagnosis and adopting a thorough approach when symptoms are unusual or worsen, especially when initial treatments do not resolve the issue [7-8]. Timely recognition and targeted treatment enabled successful management through surgical removal of the adrenal tumor, resulting in a positive outcome [9-10]. This report underscores the importance of clinical vigilance and the need for a detailed investigation when encountering rare conditions like pheochromocytoma, even when initial symptoms may suggest more common diagnoses.

## Case presentation

A 28-year-old woman with no significant medical history initially presented with intermittent palpitations that had been ongoing for two months. She described these episodes as sudden and irregular heartbeats but denied any associated symptoms such as chest pain, shortness of breath, or syncope. An initial electrocardiogram (ECG) showed sinus rhythm with occasional premature ventricular contractions (PVCs), which were considered benign given the absence of other concerning signs or risk factors. Routine blood tests, including a complete blood count (CBC) and thyroid function tests (TFTs), were normal. Based on her age and lack of underlying risk factors, she was diagnosed with likely benign ventricular ectopy and started on metoprolol 25 mg twice daily. This provided partial, temporary relief.

However, two months later, she returned with worsening symptoms. She reported more frequent and severe palpitations, now occurring at rest, along with new symptoms including night sweats and unintended weight loss of 5 kg over a few weeks. On examination, her heart rate was elevated at 120 beats per minute (bpm), and her blood pressure (BP) was significantly high at 160/100 mmHg. Apart from this, her physical exam was unremarkable, with no signs of heart failure or other abnormalities.

**Figure 1:**
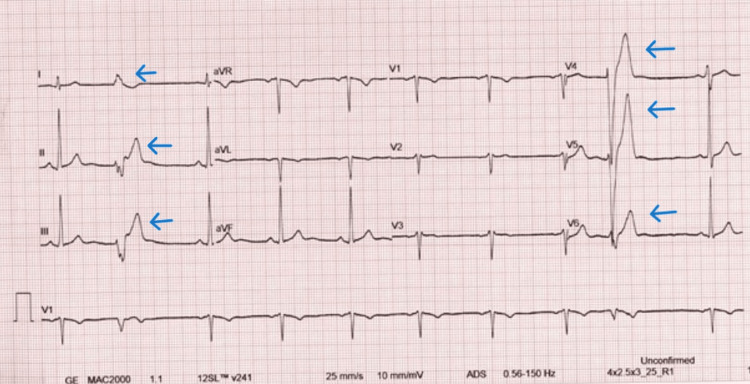
ECG showing a nonsustained ventricular tachycardia (NSVT) beats (blue arrows) ECG: Electrocardiogram

Given the worsening symptoms, a more thorough evaluation was conducted. A repeat ECG showed episodes of nonsustained ventricular tachycardia (NSVT), raising concern for a more serious underlying cause (Figure 1).

Suspecting a secondary condition, especially with the new onset of hypertension and systemic symptoms, additional tests were performed. These revealed significantly elevated levels of plasma and urinary metanephrine and normetanephrine levels, suggesting excess catecholamine production (Table 1).

**Table 1:** Lab reports

Lab reports	Results	Reference range
White blood cell count (1000/cmm)	7.1	4-11
Platelet count (1000/cmm)	191	150-450
Red blood cell count (million/uL)	3.89	4.0-5.1
Hemoglobin (g/dL)	10.5	12-14
Mean corpuscular volume (fL)	80.5	80-100
Mean corpuscular hemoglobin (pg)	30.5	27.5-33.2
Mean corpuscular hemoglobin concentration (gm/dL)	33.4	33.4-35.5
Plasma metanephrines (nmol/L)	11.7	<0.5
Plasma normetanephrines (nmol/L)	22.5	<0.9
Urinary metanephrines (ug/24h)	20	<340
Urinary normetanephrines (ug/24h)	07	<440
Thyroid stimulating hormone (TSH) (mIU/L)	3.2	0.5-5.0
Total T3 levels (ng/dL)	180	80-220
Total T4 levels (mcg/dL)	7.0	5-12
Sodium (mEq/L)	141	135-145
Potassium (mEq/L)	3.8	3.5-5.5
Magnesium (mg/dL)	1.8	1.7-2.2

**Figure 2:**
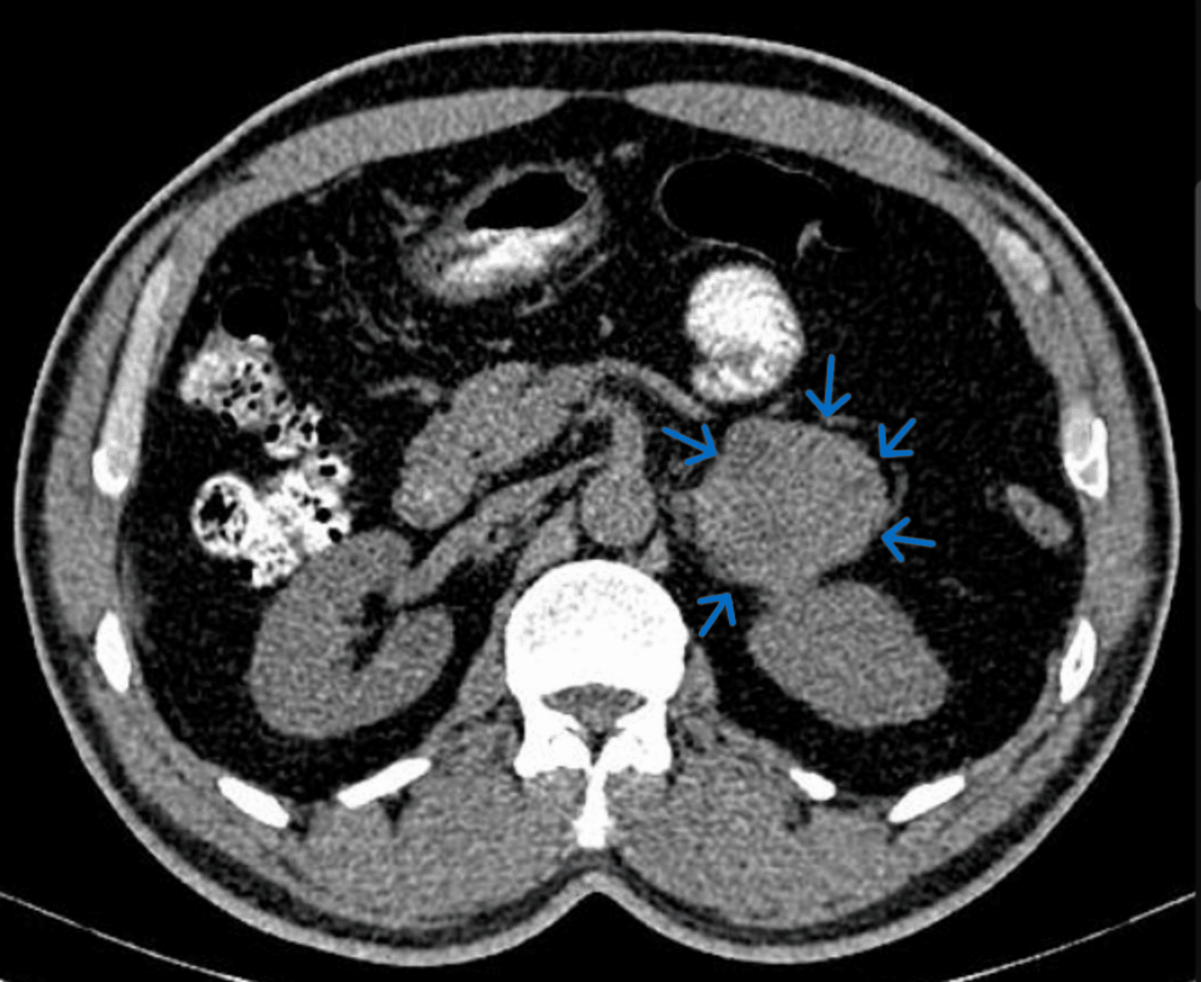
Abdominal CT scan image showing an adrenal gland mass (blue arrows) CT: Computed tomography

Further testing with a computed tomography (CT) scan confirmed the presence of a heterogeneous mass measuring 4 cm (Figure 2), which, along with the elevated metanephrines, strongly suggested a pheochromocytoma, an adrenal tumor that causes excessive catecholamine secretion, leading to symptoms like palpitations, hypertension, and arrhythmias.

Given the complexity of her condition and the potential for life-threatening cardiovascular complications, the patient was referred to a tertiary care center for further evaluation and management.

## Discussion

This case illustrates the significant diagnostic challenges encountered when a young woman presents with atypical and evolving symptoms. Initially, her intermittent palpitations seemed to align with the profile of a benign arrhythmia, particularly given her young age, overall good health, and absence of significant cardiovascular risk factors. Paroxysmal palpitations in young patients are often attributed to idiopathic causes, and early assessments, including an ECG showing occasional PVCs, did little to suggest a more serious underlying pathology. The initial treatment with metoprolol provided temporary relief, further supporting the presumption of a benign arrhythmia. However, as her symptoms persisted and began to escalate, it became clear that a more in-depth evaluation was warranted.

Over time, her palpitations increased in frequency and intensity, and she developed alarming systemic symptoms, including profuse sweating, particularly at night, and significant unintended weight loss of 5 kg over a short period. These additional symptoms indicated that her condition was not as straightforward as initially suspected. While idiopathic VT is commonly seen in young individuals without structural heart disease and was initially considered, the new symptoms suggested the possibility of a more serious underlying condition [1]. 

The differential diagnosis was systematically expanded to explore potential causes beyond benign arrhythmias. Structural heart diseases such as cardiomyopathy or ischemic heart disease, which can cause arrhythmias, were considered but ruled out by her normal echocardiogram and cardiac magnetic resonance imaging (MRI). These imaging studies confirmed that her heart was structurally sound, further diminishing the likelihood of heart disease as the root cause of her symptoms. 

Other potential causes of VT, such as electrolyte imbalances, were excluded based on her normal laboratory results. Congenital long QT syndrome, which predisposes individuals to arrhythmias and can be inherited, was also considered [4-5]. However, her ECG showed no prolongation of the QT interval, making this diagnosis less likely. Arrhythmogenic right ventricular cardiomyopathy (ARVC), a genetic condition that affects the right ventricle and can cause arrhythmias, was also ruled out by a cardiac MRI that demonstrated no abnormalities in the right ventricle.

With cardiac and metabolic causes largely ruled out, attention shifted to systemic conditions that could explain both the cardiac arrhythmias and her new symptoms of sweating and weight loss. Thyrotoxicosis, a condition in which excessive thyroid hormone levels can lead to palpitations, weight loss, and sweating, was considered. However, her normal TFTs excluded this possibility. Anxiety and panic disorders, which often manifest with palpitations and sweating, were also explored as potential causes. However, the absence of significant psychological distress and the presence of severe, physiological symptoms such as elevated heart rate and hypertension made a psychological cause less likely. Moreover, the discovery of elevated plasma free metanephrines suggested a more physiological etiology, specifically related to excess catecholamine production.

This led clinicians to suspect a catecholamine-secreting tumor, such as a pheochromocytoma or a paraganglioma. Both conditions involve neuroendocrine tumors that secrete catecholamines, which can cause a variety of symptoms, including hypertension, palpitations, sweating, and weight loss [10]. Elevated plasma free metanephrines and normetanephrines provided strong biochemical evidence supporting this suspicion. An abdominal ultrasound was performed, followed by a CT scan, which identified a 4 cm heterogeneous mass located in the left adrenal gland. This imaging finding, combined with the elevated metanephrine levels, confirmed the diagnosis of pheochromocytoma, a rare but potentially life-threatening adrenal tumor.

Pheochromocytomas are rare neuroendocrine tumors that arise from the adrenal medulla and secrete excess catecholamines, such as adrenaline and noradrenaline, which are responsible for the patient's symptoms of palpitations, hypertension, sweating, and weight loss. These tumors, though uncommon, can lead to severe cardiovascular complications if not promptly diagnosed and treated [7-8]. The combination of biochemical testing, imaging, and clinical presentation made the diagnosis clear in this case. 

This case highlights several key points in medical diagnosis and management. First, it serves as a reminder that even when a patient presents with symptoms that appear to fit a common condition, such as benign arrhythmia, clinicians must remain open to the possibility of rarer and more serious underlying conditions, especially when new or unusual symptoms emerge. Second, it emphasizes the importance of maintaining a broad differential diagnosis and the need for a comprehensive evaluation when patients do not respond as expected to initial treatments. Finally, it demonstrates the value of thorough and systematic diagnostic workups, which, in this case, ultimately led to the identification and successful treatment of a rare neuroendocrine tumor.

In summary, this young woman's clinical journey underscores the complexity of diagnosing rare conditions like pheochromocytoma, particularly when they present with nonspecific or atypical symptoms. Her case highlights the importance of persistence in clinical evaluation and the need for multidisciplinary approaches when addressing challenging diagnostic dilemmas. Through careful investigation, early diagnosis, and timely surgical intervention, this patient was able to avoid potentially life-threatening complications and achieve a positive outcome.

## Conclusions

This case report highlights the complexities of diagnosing pheochromocytoma, a rare neuroendocrine tumor, presenting as VT in a young woman. Initially misdiagnosed as benign arrhythmia due to her age and lack of risk factors, the patient’s symptoms escalated, including significant weight loss and severe sweating. Comprehensive evaluation ruled out common causes such as idiopathic VT, structural heart disease, and thyrotoxicosis, eventually revealing elevated plasma free metanephrines and an adrenal mass. The diagnosis of pheochromocytoma was confirmed by imaging and biochemical findings, leading to successful surgical intervention. This case underscores the importance of considering a wide differential diagnosis and maintaining vigilance for rare conditions, particularly when symptoms are atypical. Early recognition and targeted treatment are crucial for preventing severe complications and ensuring favorable outcomes.
